# Rapid Assessment of SARS-CoV-2 Variant-Associated Mutations in Wastewater Using Real-Time RT-PCR

**DOI:** 10.1128/spectrum.03177-22

**Published:** 2023-01-11

**Authors:** Kata Farkas, Cameron Pellett, Rachel Williams, Natasha Alex-Sanders, Irene Bassano, Mathew R. Brown, Hubert Denise, Jasmine M. S. Grimsley, Jessica L. Kevill, Mohammad S. Khalifa, Igor Pântea, Rich Story, Matthew J. Wade, Nick Woodhall, Davey L. Jones

**Affiliations:** a Centre for Environmental Biotechnology, School of Natural Sciences, Bangor University, Bangor, Gwynedd, United Kingdom; b School of Ocean Sciences, Bangor University, Menai Bridge, Anglesey, United Kingdom; c UK Health Security Agency, Environmental Monitoring for Health Protection, London, United Kingdom; d Department of Infectious Disease, Imperial College London, London, United Kingdom; e School of Engineering, Newcastle University, Newcastle-upon-Tyne, United Kingdom; f The London Data Company, London, United Kingdom; g Division of Biosciences, College of Health, Medicine and Life Sciences, Brunel University, London, United Kingdom; h Servita Professional Services (UK) Ltd., London, United Kingdom; i Food Futures Institute, Murdoch University, Murdoch, Western Australia, Australia; University of Prince Edward Island

**Keywords:** digital PCR, quarantine hotel monitoring, human health risk, single nucleotide polymorphism, airport sewage surveillance, variant of concern

## Abstract

Within months of the COVID-19 pandemic being declared on March 20, 2020, novel, more infectious variants of SARS-CoV-2 began to be detected in geospatially distinct regions of the world. With international travel being a lead cause of spread of the disease, the importance of rapidly identifying variants entering a country is critical. In this study, we utilized wastewater-based epidemiology (WBE) to monitor the presence of variants in wastewater generated in managed COVID-19 quarantine facilities for international air passengers entering the United Kingdom. Specifically, we developed multiplex reverse transcription quantitative PCR (RT-qPCR) assays for the identification of defining mutations associated with Beta (K417N), Gamma (K417T), Delta (156/157DEL), and Kappa (E154K) variants which were globally prevalent at the time of sampling (April to July 2021). The assays sporadically detected mutations associated with the Beta, Gamma, and Kappa variants in 0.7%, 2.3%, and 0.4% of all samples, respectively. The Delta variant was identified in 13.3% of samples, with peak detection rates and concentrations observed in May 2021 (24%), concurrent with its emergence in the United Kingdom. The RT-qPCR results correlated well with those from sequencing, suggesting that PCR-based detection is a good predictor for variant presence; although, inadequate probe binding may lead to false positive or negative results. Our findings suggest that WBE coupled with RT-qPCR may be used as a rapid, initial assessment to identify emerging variants at international borders and mass quarantining facilities.

**IMPORTANCE** With the global spread of COVID-19, it is essential to identify emerging variants which may be more harmful or able to escape vaccines rapidly. To date, the gold standard to assess variants circulating in communities has been the sequencing of the S gene or the whole genome of SARS-CoV-2; however, that approach is time-consuming and expensive. In this study, we developed two duplex RT-qPCR assays to detect and quantify defining mutations associated with the Beta, Gamma, Delta, and Kappa variants. The assays were validated using RNA extracts derived from wastewater samples taken at quarantine facilities. The results showed good correlation with the results of sequencing and demonstrated the emergence of the Delta variant in the United Kingdom in May 2021. The assays developed here enable the assessment of variant-specific mutations within 2 h after the RNA extract was generated which is essential for outbreak rapid response.

## INTRODUCTION

The SARS-CoV-2 and its associated disease, COVID-19, have been responsible for over 0.5 billion confirmed cases and 6.5 million deaths globally as of August 2022 (1). The rapid spread of the disease is partially a result of the emergence of novel viral variants, which may be more transmittable than the wild-type Wuhan virus. For instance, the Alpha variant (B.1.1.7 lineage), first detected in the United Kingdom in December 2020 (2), had a 1.22 to 2.49 times higher reproduction numbers than the Wuhan strain due to mutations in the angiotensin-converting enzyme 2 (ACE2) receptor-binding site of the spike protein gene (3). During the same period, additional, highly transmittable variants of concern (VOCs), associated with large numbers of cases, emerged in South Africa (Beta variant, B.1.315 lineage) and Brazil (Gamma variant, P1 lineage) (4, 5). In mid-2021, the B.1.617 lineages, including the Delta and Kappa variants, emerged in India. By April 2021, the Delta variant became the most common variant in India and surrounding countries and had spread globally. Between July and December 2021, the Delta variant was responsible for over 90% of SARS-CoV-2 infections in the United Kingdom, and also prompted new waves of COVID-19 outbreaks across Europe, Indonesia, and the Americas (6). Since December 2021, these variants have been progressively replaced by the more transmissible Omicron subvariants (7).

Approximately 40% to 45% of SARS-CoV-2 infections remain asymptomatic, and hence, are missed by clinical surveillance (8). Furthermore, the proportion of clinical reverse transcription quantitative PCR (RT-qPCR) false negative tests was estimated to be between 2% and 29% with sensitivity of only 63% for nasal and 32% for throat swabs, respectively (9, 10). Therefore, assessing the levels of infection solely on clinical testing is challenging. As approximately 43% to 54% of infected people shed SARS-CoV-2 in feces (11, 12), the viral RNA could be detected and quantified in wastewater. The changes in viral quantities in sewage can supplement outbreak surveillance at community level (13, 14). Hence, wastewater-based epidemiology (WBE) has been implemented in many countries as a supplementary monitoring tool (15–21). WBE has also been successful as an early warning system at the local infrastructure scale, including university campuses and prisons (22–24), demonstrating that WBE can be utilized for near-source monitoring. Furthermore, the usefulness of WBE for international border control has also been investigated. Preliminary studies focusing on airplane wastewater surveillance, suggested that SARS-CoV-2 can also be detected, quantified, and sequenced in such matrices (25, 26).

When WBE is applied, wastewater samples are usually clarified and concentrated to quantitatively enrich viruses (27). Subsequently, the viral RNA is extracted and quantified using RT-qPCR or RT digital PCR (RT-dPCR), targeting conserved regions of the nucleocapsid or envelope genes (19, 27–29). These assays enable the rapid detection and quantification of the target virus; however, they do not indicate the presence of variant of concerns (VOCs). Variant analysis is predominantly done by amplifying fragments of the viral genome from RNA extracts, followed by sequencing (30). Sequencing and data analysis may take several days, leading to delayed outbreak response. Therefore, rapid qPCR assays targeting variant-specific mutations have been developed and used for WBE in Israel (31, 32), Spain (33), and Canada (34) for example. However, variant-level qPCR detection has not been rigorously tested in near-source wastewater environments or for international border surveillance.

In this study, we describe two duplex RT-qPCR assays for the targeted detection of variant-specific mutations of the Beta, Gamma, Delta, and Kappa variants for tracking infections in wastewater at COVID-19 quarantine facilities associated with international travel hubs. The assays targeted point mutations or deletions in the spike protein gene specific to the aforementioned VOCs. The primer and probe sets were additionally trialed on a droplet digital PCR (ddPCR) system and the results of PCR-based detections were compared to those obtained from genome sequencing. The RT-qPCR assays were suitable for the rapid detection and quantification of the target VOCs in wastewater samples.

## RESULTS

### RT-qPCR assay validation.

Assay sensitivity and specificity were tested on a dilution series of synthetic viral RNA of the variants. Cross-reactivity was only observed for the Beta VSM in the Beta-Gamma duplex VSM assay at high viral RNA concentrations (>10^4^ gc/μL standard solution), whereas no cross-reaction was observed at lower concentrations. Cross-reactivity was further tested on historic RNA extracts from wastewater samples collected before the emergence of SARS-CoV-2 variants. These samples were negative for all target mutations. All assays were highly sensitive with LOD values of 1 to 4 gc/μL, whereas the LOQ values were lower for the Beta and Gamma VSMs and higher for the Delta and Kappa VSMs (Table 1).

**TABLE 1:** Limit of detection (LOD) and limit of quantification (LOQ) values for each SARS-CoV-2 variant target determined in duplex RT-qPCRs expressed as genome copies (gc) in standard solution

Variant	LOD (gc/μL)	LOQ (gc/μL)
Beta	1.04	4.28
Gamma	0.75	4.40
Delta	1.88	25.72
Kappa	2.95	15.77

### RT-qPCR assay applicability for wastewater samples in near-source setting.

**FIG 1:**
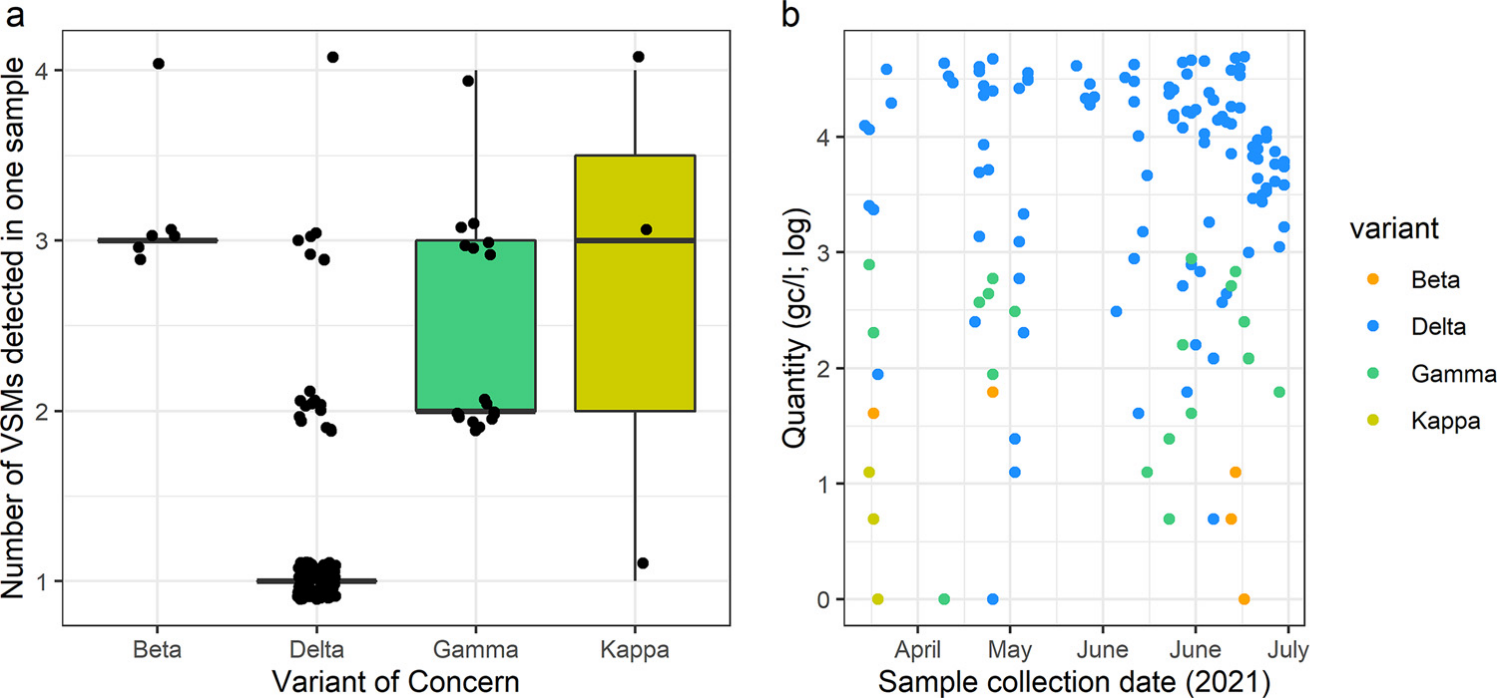
Variant-specific mutations (VSMs) associated with the Beta (orange), Gamma (green), Delta (blue), and Kappa (yellow) variants of concern (VOC) in wastewater using RT-qPCR. Panel (a) shows the codetection rates for the VSMs and panel (b) shows the viral concentrations (log_10_ genome copies/ L) over time.

Wastewater monitoring was carried out at 13 hotels used for quarantining international air passengers entering the United Kingdom during the emergence of the Delta variant. Out of the 820 sewage sample extracts tested, 459 were positive for the SARS-CoV-2 N1 gene target. All 820 samples were tested for the Beta and Gamma VSMs while 818 samples (as two samples were destroyed) were tested for the Delta and Kappa VSMs using two separate duplex RT-qPCR assays (Fig. 1). In total, 110 samples were positive for at least one variant, 19 were found positive for at least two variants, seven were positive for at least three variants, while one was found positive for all four variants (Fig. 1a). Interestingly, the Beta VSM was only detected by RT-qPCR in the presence of the Gamma VSM (n = 6), and all detections of the Beta VSM were at high concentrations of the SARS-CoV-2 N1 gene fragment. The most detected VSM was Delta (109 positives, 13.3%), followed by Gamma (19 positives, 2.3%), Beta (9 positives, 0.7%) and Kappa VSMs (3 positives, 0.4%). Almost all samples which tested positive for the VSMs were positive for the N1 gene, except one Delta VSM positive sample. The Kappa VSM was only detected in samples collected in April 2021 (2.5%), whereas the Beta, Gamma, and Delta VSMs were more abundant throughout the study, with peak detections in May and lower concentrations in June to July (Table 2).

**TABLE 2:** Temporal changes in the detection frequency of the Beta, Gamma, Delta, and Kappa VSMs in wastewater from quarantining hotels using RT-qPCR

Sampling date	SARS-CoV-2 (all variants)^*a*^	Beta	Gamma	Delta	Kappa
April 2021	56.1% (69/123)	0.8% (1/123)	1.6% (2/123)	5.8% (7/121)	2.5% (3/121)
May 2021	79.8% (83/104)	1.9% (2/104)	5.8% (6/104)	24.0% (25/104)	0% (0/104)
June 2021	50.6% (172/342)	0% (0/342)	1.8% (6/341)	10.2% (35/342)	0% (0/342)
July 2021	53.0% (133/251)	1.2% (3/251)	2.0% (5/251)	16.7% (42/251)	0% (0/251)

a The values in parentheses denotes the number of positives relative to the total number of samples.

To verify the VSM RT-qPCR results, they were compared with the next generation sequencing (NGS) data (Table S1). NGS and RT-qPCR data for the Beta, Delta, and Kappa variants were available for the period of April 13, 2021 to June 18, 2021. At least one variant was detected in 307 samples, and two variants were detected in 34 samples using NGS analysis. Only 13 samples were positive for the Beta variant from the sequencing results; however, none of those samples were found positive using RT-qPCR. For the Delta variant, 270 samples were NGS-positives, and of those samples, 89 were also positive using RT-qPCR. The Kappa variant was positive in 35 samples that were sequenced, two of which were RT-qPCR positive. Among the samples that tested negative with NGS, but positive with RT-qPCR, six were positive for the Beta variant, 19 for the Delta variant, 19 for the Gamma variant, and one for the Kappa variant. Using logistic regression, RT-qPCR detections of VSMs were found to be a significant predictor of NGS VOC detection (*P*-value < 0.001; Table S1), though use of RT-qPCR quantities rather than RT-qPCR detection did not improve the misclassification error (15.1%).

### RT-ddPCR assay applicability for wastewater samples in near-source setting.

The in-house designed oligos for qPCR-based detection of VSMs were first trialed on a ddPCR platform using known concentrations of SARS-CoV-2 RNA or DNA standards. We found that the RT-ddPCR gave similar results to RT-qPCR when DNA target oligos were used; however, it was less sensitive with RNA targets (synthetic viral genomes). Furthermore, ddPCR failed to detect the Kappa VSMs using DNA oligo as a target (Fig. S2).

We further tested the usefulness of ddPCR for detecting variants from sewage samples. We used RT-ddPCR with the in-house designed primers and probes on samples that were RT-qPCR and/or NGS positive for variants. Overall, ddPCR resulted in higher detection rates than qPCR, with limited samples testing positive for the variants by both approaches (Table 3).

**TABLE 3:** Number of samples tested positive with qPCR and ddPCR only and with both assays

Target	n	Positive with qPCR	Positive with ddPCR	Positive with qPCR and ddPCR
Beta VSM	14	1	5	3
Gamma VSM	22	3	12	6
Delta VSM	23	1	9	9
Kappa VSM	6	0	0	0

**FIG 2:**
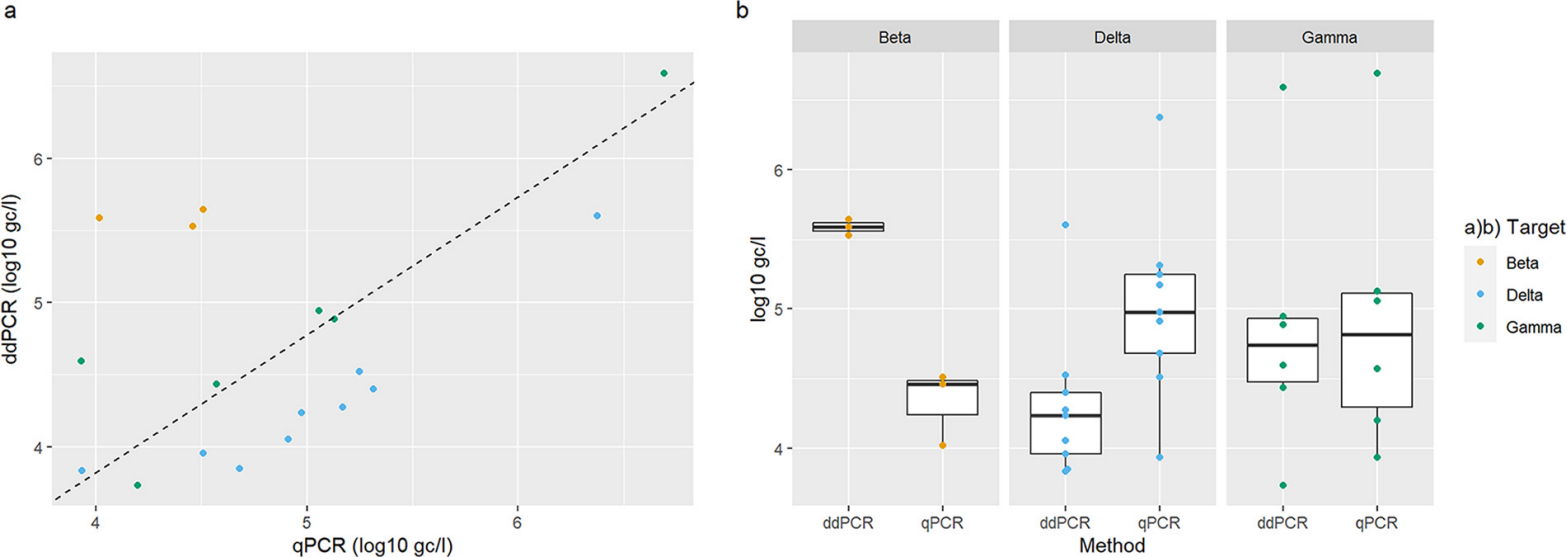
Quantification and detection of the Beta, Gamma, Delta, and Kappa VSMs in wastewater with RT-qPCR and RT-ddPCR. Panel (a) shows the correlation between quantities of VSMs determined with RT-ddPCR and RT-qPCR (Pearson's product-moment correlation: *t* = 2.3, df = 16, *P*-value < 0.05), with the dashed line fitted using linear regression. Panel (b) shows the difference between absolute quantities determined using both PCR methods.

The concentrations of Delta and Gamma VSMs determined with RT-ddPCR and RT-qPCR correlated closely (Fig. 2a; *P*-value < 0.001), but concentrations of Beta VSMs did not. The concentrations of the VSMs determined by RT-ddPCR were higher (Beta median: 5.59 log_10_ gc/l; Gamma median: 4.74 log_10_ gc/l; Delta median: 4.24 log_10_ gc/l) than the concentrations determined by RT-qPCR (Beta median: 4.46 log_10_ gc/l; Gamma median: 4.81 log_10_ gc/l; Delta median: 4.97 log_10_ gc/l), although the differences were not significant (Fig. 2b; Wilcoxon rank sum exact test: (Beta) W = 6, *P*-value > 0.05; (Gamma) W = 16, *P*-value > 0.05; (Delta) W = 15, *P*-value > 0.05). More samples were positive for VSMs when using RT-ddPCR (n = 26) compared to RT-qPCR (n = 22); however, qPCR assays outperformed ddPCR for the detection and quantification of the Delta VSM (Fig. 2; Fig. S1). RT-ddPCR had greater agreement with NGS (Fig. S1; Table S2; RT-ddPCR misclassification error: 52.4%; qPCR misclass. error: 85.7%). However, with this subset of data, neither PCR methods were significant predictors of NGS (*P*-value > 0.05), which may be due RT-ddPCR testing not being carried out on samples with negative results for both NGS and RT-qPCR. Additionally, the six samples positive for the Kappa VSMs using NGS were negative using PCR-based quantification.

## DISCUSSION

In this study, we developed and validated novel RT-qPCR assays for the detection of emerging SARS-CoV-2 variants. Since the Alpha, and subsequently other VOCs were identified, many laboratories have been designing PCR-based assays for the rapid identification of new threats (Table 4). Most efforts have focused on defining mutations specific to the Alpha variant, such as the 69-70DEL and 144DEL (34, 49–56). Assays are also available for mutations common to the Alpha, Beta, and Gamma variants, such as the N501Y and E484K SNPs (34, 49, 52, 54, 55, 57, 58). Only a few studies have described qPCR-based assays for the individual detection of Beta, Gamma, and Delta variants (31, 51, 52, 55, 59) that were circulating in the United Kingdom in the early half of 2021.

**TABLE 4:** qPCR assays developed for SARS-CoV-2 variants^*a*^

Reference	Application	Target variant	Lineages	Target VSM
49	Clinical	Alpha	B.1.1.7	69-70DEL
		Alpha/Beta/Gamma/Omicron/Mu	B.1.1.7; B.1.351; P1; B.1.617.2; BA.1-2; BA.4-5; BA.2.12.1; B.1.621	N501Y
50	Clinical	Alpha	B.1.1.7	69-70DEL
59	Clinical	Alpha	B.1.1.7	D3L^*b*^
		Beta	B.1.351	242-244DEL
53	Clinical	Alpha	B.1.1.7	69-70DEL; 144DEL
60	Clinical	Omicron	BA.1	211-214INSDEL
54	Clinical	Alpha	B.1.1.7	69-70DEL
		Alpha/Beta/Gamma/Omicron/Mu	B.1.1.7; B.1.351; P1; B.1.617.2; BA.1-2; BA.4-5; BA.2.12.1; B.1.621	N501Y
		Beta/Gamma/Eta/Iota/Mu	B.1.351; P1; B.1.525; B.1.526; B.1.621	E484K
52	Clinical	Alpha	B.1.1.7	69-70DEL
		Alpha/Beta/Gamma/Omicron/Mu	B.1.1.7; B.1.351; P1; B.1.617.2; BA.1-2; BA.4-5; BA.2.12.1; B.1.621	N501Y
		Beta/Gamma/Eta/Iota/Mu	B.1.351; P1; B.1.525; B.1.526; B.1.621	E484K
		Beta	B.1.351	K417N
55	Clinical	Alpha	B.1.1.7	69-70DEL
		Alpha/Beta/Gamma/Omicron/Mu	B.1.1.7; B.1.351; P1; B.1.617.2; BA.1-2; BA.4-5; BA.2.12.1; B.1.621	N501Y
		Beta/Gamma/Eta/Iota/Mu	B.1.351; P1; B.1.525; B.1.526; B.1.621	E484K
		Beta	B.1.351	K417N
		Kappa/Delta	B.1.617.1; B.1.617.2	L452R
		Delta/Omicron	B.1.617.2; BA.1-2; BA.4-5; BA.2.12.1	T478K
57	Clinical	Alpha/Beta/Gamma/Omicron/Mu	B.1.1.7; B.1.351; P1; B.1.617.2; BA.1-2; BA.4-5; BA.2.12.1; B.1.621	N501Y
58	Clinical	Alpha/Beta/Gamma/ Omicron/Mu	B.1.1.7; B.1.351; P1; B.1.617.2; BA.1-2; BA.4-5; BA.2.12.1; B.1.621	N501Y
61	Clinical	Omicron	BA.1-2; BA.4-5; BA.2.12.1	S477N
51	Wastewater	Alpha	B.1.1.7	69-70DEL
		Beta	B.1.351	241-243DEL
31	Wastewater	Delta	B.1.617.2	157-158DEL
		Gamma	P1	28269-28273INS^*c*^
34	Wastewater	Alpha	B.1.1.7	69-70DEL; D3L^*b*^
		Alpha/Beta/Gamma/ Omicron/Mu	B.1.1.7; B.1.351; P1; B.1.617.2; BA.1-2; BA.4-5; BA.2.12.1; B.1.621	N501Y
56	Wastewater	Alpha	B.1.1.7	69-70DEL; 144DEL; A570D

a Variant and lineage information were adopted from https://covariants.org/, as accessed on the May 31, 2022. VSM indicate point mutations, insertions (INS), and deletions (DEL) in the amino acids of the S protein gene, unless stated otherwise.

b Mutation in N gene.

c Four-nucleotide insertion in ORF8.

In this study, we developed qPCR-based assays for quantitatively detecting VOC-specific SNPs for the Beta, Gamma, and Kappa SARS-CoV-2 variants and a deletion specific to the Delta VOC by the time the study was conducted. Yaniv et al. also designed a qPCR assay within the same region of the Delta genome that we targeted and used the assay to successfully identify Delta in wastewater samples. To our knowledge, the method described in this paper is the first qPCR assay selective for the Kappa variant. For the Beta and Gamma VSMs, we targeted one SNP site responsible for the amino acid changes of K417N and K417T, respectively. Previous studies have attempted to target this SNP leading to K417N SNP using qPCR with a custom-designed or commercial primer/probe sets (52, 55). However, these assays were tested only in clinical settings, where the samples were derived from one patient and hence likely to represent one genome.

In our study, the assays were performed using wastewater samples from quarantine facilities, where they were expected to contain multiple SARS-CoV-2 lineages. We found that the Beta VSM was detected using RT-qPCR only when the concentrations of SARS-CoV-2 were high and when the Gamma VSM was also detected, which may suggest that the assay was not specific to the SNP K417N or that the SNP may occur in other, less transmittable lineages not identified by the time of the study. However, it is also possible that the point mutation was introduced during the extension process of the PCR assay due to polymerase base substitution errors (62, 63). Therefore, when the K417N mutation is detected using qPCR, the results should be verified by targeting another VSM using qPCR (56) or by sequencing.

We also assessed the usefulness of ddPCR for variant detection using synthetic RNA and a subset of wastewater samples. We found high detection rates for three out of four VSMs and a good correlation between qPCR/ddPCR and NGS results, suggesting that this approach could be applicable for detecting SARS-CoV-2 and possibly other viruses in wastewater. Previous research has also found good correlation between ddPCR and targeted amplicon sequencing for the detection of SARS-CoV-2 in wastewater samples (64). Comparative studies suggested that ddPCR may be superior to qPCR for SARS-CoV-2 quantification in wastewater (65, 66). However, due to limitations of the reagents used for reverse transcription and amplification, ddPCR could be less sensitive than qPCR-based detection for some targets. As up to 10 μL of RNA extract can be assayed (compared to the maximum of 4 to 5 μL in qPCR), this limitation could be overcome. Nonetheless, the ddPCR approach is more expensive and time-consuming than qPCR (Table 5) and would require more validation and optimization to gain reliable results.

**TABLE 5:** Comparison of the qPCR and ddPCR approaches for SARS-CoV-2 detection

Criterium	qPCR	ddPCR
Platform tested	QuantStudio Flex 6 384 platform (Applied Biosystems, Inc., USA)	QX200 Droplet Digital PCR System (Bio-Rad Laboratories, USA)
Samples / run	184	94
Run time	1.5 hours (1 step)	1 day (3 steps)
Quantification type	Relative to standards	Absolute
Quantification range	3 to 1,000,000 copies/reaction	5 to 15,000 copies/reaction
Equipment cost	£37,000	£110,000
Reagent cost/90 samples	£445	£520 to 600
Sample vol/reaction	4 μL	10 μL
Amplicon sequencing	Yes	No

In this study, we implemented WBE for monitoring COVID-19 in hotels used as temporary accommodation for people travelling to the United Kingdom from countries with high COVID prevalence. Travelers were expected to stay for 10 days in the hotels and take two COVID-19 PCR tests on days 2 and 8. We conducted wastewater testing at the early stages of the third COVID-19 wave due to the emergence of the Delta variant in April to July 2021. As expected, the majority (56%) of the wastewater samples tested positive for SARS-CoV-2. Using RT-qPCR, we sporadically detected defining mutations associated with the Beta, Gamma, and Kappa variants. The VSM associated with the Delta variant was commonly detected since May 2021, which coincided with the emergence of the Delta variant in the United Kingdom (67). Sequencing-based detection also identified the target variants, and a strong correlation between the variants identified using qPCR and sequencing was found. However, the deletion targeted by qPCR to detect the Delta variant fell in the overlap between amplicons of the used primer scheme for sequencing, thus, a direct comparison of its detection between the two approaches was not possible.

To date, this is the first study using WBE in a quarantine facility in the context of border surveillance. Our results suggest that PCR-based VSM detection is a good predictor for variant presence in wastewater samples. However, due to the nature of PCR-based detection, false identification may occur, thus positive samples can be used to determine whether further tests and sampling is necessary. This approach supports the timely identification of SARS-CoV-2 variants among people entering the United Kingdom, given that sampling can be done daily without invasive or sampling bias. The use of qPCR-based VSM detection further reduced analysis time to a few hours, as opposed to several days required for sequencing (including library preparation, sequencing run, data analysis, and interpretation). Overall, qPCR results on VSMs may be available within 24 to 48 h postsampling, depending on the length of the wastewater processing methods. In conclusion, we have shown that wastewater-based RT-qPCR-based assays can be readily deployed to track the entry of different variants of SARS-CoV-2 across international borders and to validate the usefulness of travel quarantining facilities.

## MATERIALS AND METHODS

### Primer and probe design.

Reference sequences for the Beta (EPI_ISL_678597), Gamma (EPI_ISL_792683), Delta (EPI_ISL_1544014), and Kappa (EPI_ISL_1662307) SARS-CoV-2 variant genomes were taken from the GISAID database (35). The probes were designed to target-defining mutations of each variant, as detailed in Table 6, which were identified using Nextstrain resources (36). The primers and probes were designed using Geneious Prime v2021.1.1 (Biomatters, New Zealand). For each target, two to four primers and one to two probes were designed. All oligos were tested in different combinations, and the most sensitive assays were selected for further testing (Table 6). To enable duplexing, the probes targeting the Gamma and Delta VSMs were labeled with FAM as a reporter, whereas the Beta and Kappa probes were labeled with HEX. The primers and probes were purchased from Integrated DNA Technologies (IDT; USA) and Eurogentec S.A. (Belgium).

**TABLE 6:** Summary of the primers and probes designed to target variant-specific mutations (VSM) of the Beta, Gamma, Delta, and Kappa variants of SARS-CoV-2

Target variant	Target VSM	Oligo type	Sequence^*a*^
Beta (B.1.315)	K417N	Forward primer	TGAAGTCAGACAAATCGCTCC
		Reverse primer	CAAGCTATAACGCAGCCTGT
		Probe	**HEX**-AGGGCAAACTGGAAATATTGCTG-**BHQ**
Gamma (P1)	K417T	Forward primer	TGAAGTCAGACAAATCGCTCC
		Reverse primer	CAAGCTATAACGCAGCCTGT
		Probe	**FAM**-ACTGGAACGATTGCTGATTATAATT-**MGB**
Delta (B.1.617.2)	156-157DEL	Forward primer	GATCCATTTTTGGGTGTTTATTACC
		Reverse primer	GGCTGAGAGACATATTCAAAAGTG
		Probe	**FAM**-TGGAAAGTAGAGTTTATTCTAGTGCG-**MGB**
Kappa (B.1.617.1) including B.1.617.3)	E154K	Forward primer	GCCGGTAGCACACCTTGTAA
		Reverse primer	GTTGGAAACCATATGATTGTAAAGGA
		Probe	**HEX**-TGGTGTTCAAGGTTTTAATTGTTAC-**BHQ**

a The dye names are present in bold.

### RT-qPCR assay.

The RT-qPCR assays were carried out using the QuantStudio Flex 6 real-time PCR system (Applied Biosystems, USA). Each 20 μL reaction mix contained 1× TaqMan Fast Virus 1-Step Master Mix (Applied Biosystems, USA) with 10 pmol forward primer, 20 pmol reverse primer, 5 pmol probe, 16 nmol MgSO_4_, 1 μg bovine serum albumin (BSA), and 4 μL sample/standard/control. Amplification was carried out using the following thermal cycling conditions: reverse transcription at 50°C for 30 min followed by enzyme inactivation at 95°C for 20 s, then 45 amplification cycles of 95°C for 3 s, 55°C/58°C/60°C for 30 s. Our preliminary data showed that the assays performed the best with annealing/extension at 58°C; hence, this temperature was used in subsequent reactions.

Each reaction plate contained two to four nontemplate controls (NTCs), where molecular grade water was added to the reaction mix instead of samples to confirm the absence of contamination. We used a serial dilution of standards with a concentration range of 10° to 10^5^ genome copies (gc)/μL of standard in duplicate for method development and quantification. We used commercially available synthetic RNA standards for the Beta, Gamma, and Delta variants (Standard 16-17-18, Twist Bioscience, USA) which were identical to the reference genomes used for primer and probe design. Due to the lack of commercially available genome standards, we used synthetic DNA incorporating the target sequence, based on the VoC reference genome (detailed above), for the Kappa variant (IDT, USA).

After each run, the threshold values were manually adjusted when the noise levels were high. The standard curves met the criteria described in the MIQE guidelines (37), with the slope and efficiency being within recommended limits of −3.1 to 3.6 and 90% to 110%, respectively, as detailed in Table 7. The sample RNA concentrations were calculated using the QuantStudio Flex 6 Real-Time PCR software v1.7 and expressed as gc/μL RNA extract. The virus RNA concentration in the wastewater samples were calculated as:

$$\text{Wastewater virus concentration}\;(\text{gc}/\text{l})=\frac{\text{concentration of the RNA extract}\left(\frac{\text{gc}}{\text{μl}}\right)\times \text{RNA extract volume}\;\left(0.1\;\text{ml}\right)}{\text{volume of sample processed}\;\left(150\;\text{ml}\right)}*1000\;\text{ml}$$

**TABLE 7:** Standard curve slope, efficiency, and R^2^ values for each target variant-specific mutation of SARS-CoV-2

Target	Slope	R^2^	Efficiency %
Beta	–3.1	0.999	110
Gamma	–3.2	0.983	107
Delta	–3.6 to –3.3	0.957 to 0.998	92 to 103
Kappa	–3.1 to –3.2	0.987 to 0.998	108 to 109

### RT-ddPCR.

RT-ddPCR assays were carried out using the QX200 AutoDG Droplet Digital PCR System (Bio-Rad, USA). The 20 μL reaction mix, containing 1× One-step RT-ddPCR Super Mix, 1× One-step RT-ddPCR Reverse transcriptase, 15 mM DTT, 10 pmol forward primer, 20 pmol reverse primer, 5 pmol probe, and 4 μL wastewater extract or standard was subject to automated droplet generation. The resulting mixture was subject to PCR amplification with the following reaction conditions: reverse transcription at 50°C for 60 min, inactivation at 95°C for 10 min, 40 cycles of denaturation at 95°C for 30 s and annealing-extension at 58°C for 4 min, followed by deactivation at 98°C for 10 min, and hold at 4°C. The concentrations were determined using a QX2000 Droplet reader. The optimal annealing-extension temperature was determined using RNA/DNA standards with concentrations of approximately 500 gc/μL with 55°C to 65°C gradient.

### Limit of detection and limit of quantification.

We tested the limit of detection (LOD) and limit of quantification (LOQ) for the two duplex RT-qPCR assays targeting the Beta/Gamma and Delta/Kappa variants by spiking wastewater extracts with viral RNA standards (Twist Bioscience, USA) at nominal concentrations of 100, 50, 20, 10, 5, and 2 gc/μL. Ten replicates of each dilution were then tested and quantified using a dilution series of RNA/DNA standards, detailed above. The LOD was determined as the lowest concentration where all 10 replicates were positive and the LOQ was determined as the lowest concentration where the coefficient of variance was below 0.25 (38).

### Assessment of cross-reactivity.

To assess whether the assays are specific for the target VSMs, each primer and probe set was tested using RT-qPCR assays on dilution series of RNA from the Wuhan strain, the Alpha, Beta, Gamma, Delta, and Kappa variants. For all variants, we used synthetic RNA (Twist Bioscience, USA). For the Wuhan strain and the Alpha, Beta and Delta variants, we also used RNA extracts from *in vitro* cultured and heat-inactivated SARS-CoV-2, kindly provided by Richard Stanton (Cardiff University, UK).

### Wastewater sample collection and process.

The duplex RT-qPCR assays were tested on composite wastewater samples taken daily at 13 managed quarantine facilities as part of the English COVID-19 wastewater surveillance program (21). These facilities constituted large hotels adjacent to international air travel hubs where passengers were placed into self-isolation for 8 days upon entry to the United Kingdom. The sampling team were able to confirm at each location that all laundry and industrial cleaning services took place off-site, thus reducing the risk of sample contamination and/or signal reduction.

Samples of wastewater were taken from either the main sewer drain leaving each hotel, or from sumps/pumping stations where access to a main sewer drain was not viable. Sampling weas conducted using diurnal refrigerated composite autosamplers between April 1, 2021 and July 23, 2021 at Sites 1 to 5; between April 1, 2021 and July 19, 2021 at Sites 6 to 9, and between June 12, 2021 and July 23, 2021 at Sites 10 to 13. The samplers were configured to draw 250 mL of wastewater every 15 min over a 24-h period at each sampling location. In total, 820 samples were collected. Samples were transported at 4°C within 24 h to the laboratory, spiked with phi6 bacteriophage (process control virus) and concentrated using ammonium sulfate precipitation, followed by NucliSens extraction reagents (bioMérieux, France) as described elsewhere (39). The extracted samples were tested for phi6 concentrations to assess viral recovery in each sample (39) and for the N1 fraction of the SARS-CoV-2 genome (28, 40) prior to VSM RT-qPCRs.

### Historic sample analysis.

To demonstrate specificity, RNA extracts from 12 wastewater samples taken at six large centralized urban wastewater treatment sites on the weeks commencing April 14, 2020 and May 18, 2020 were also tested. The samples were concentrated using ultrafiltration and extracted as described previously (41). The N1-specific RT-qPCR assays suggested that 10 samples were positive for SARS-CoV-2 with concentrations between 1 and 629 genome copies (gc)/μL RNA extract.

### Next-generation sequencing.

Tiled amplicon sequencing libraries were generated from extracted samples using the EasySeq SARS-CoV-2 WGS Library Prep kit (NimaGen, the Netherlands) using Nimagen V2 (February 2021 to May 2021) and V3 (May 2021 to January 2022) primer schemes. Sequencing was performed as described elsewhere (42). In brief, the method contained three sections: (i) clean up using AMPure RNA XP beads (Beckman Coulter Agencourt) or Mag-Bind TotalPure NGS beads (Omega Bio-Tek); (ii) reverse-transcription using the LunaScript RT SuperMix kit (New England Biolabs); and (iii) reverse complement PCR (RC-PCR) using the EasySeq RC-PCR SARS-CoV-2 WGS kit (NimaGen). Amplicons were then pooled, and libraries purified with Mag-Bind (T) Total Pure NGS beads (Omega Bio-Tek) before sequencing on an Illumina NovaSeq 6000 (2 × 150 bp) at the University of Liverpool and Exeter sequencing centers or on an Illumina NextSeq 500 (2 × 150 bp) at the University of Nottingham sequencing center.

Raw reads were processed following the ARTIC pipeline (ncov2019-artic-nf; Illumina workflow; https://artic.network/ncov-2019/ncov2019-bioinformatics-sop.html. First, amplicon reads were filtered using Trim Galore v0.6.5 (https://github.com/FelixKrueger/TrimGalore) and then mapped to the reference SARS-CoV-2 genome (NCBI GenBank Accession MN908947.3) (43) using BWA v0.7.17 (44). Adapter trimming was performed using iVar v1.3 and bed files containing the genome positions of the 154 primers used to generate the amplicons (Nimagen V2 and V3 primer schemes). The resulting BAM files were sorted and indexed using SAMtools v1.13 (45) and then were submitted to VarScan v2.4.4 (46) to identify SNPs and InDels. In parallel, the indexed Bam files were submitted to COJAC (47) to identify cooccurring SNPs on the same amplicon. An in-house python script was then used to match SNP profiles and cooccurring SNPs to SARS-CoV-2 variant definitions provided by PHE (https://github.com/phe-genomics/variant_definitions). The output was a list of the variants detected in each wastewater sample. A visual review was performed to add a “Confirmed,” “Possible,” or “Not detected” status to each detection, reflecting how closely the profile matched the variant definition. Here, a sample was considered positive for a variant when both a “Confirmed” or “Possible” detection was assigned.

### Data analysis.

RT-qPCR assays were analyzed using the QuantStudio Real-Time PCR Software v1.7.2 (Applied Biosystems, Waltham, USA). RT-ddPCR assays were analyzed using Bio-Rad QX One c1.2 software (Bio-Rad Laboratories Ltd., UK). Exported quantification values were analyzed in R v4.1.2 (48) utilizing packages “readxl” and “tidyverse.” PCR methods for detecting VSMs were compared with NGS using generalized linear models with binomial residuals and evaluated by parameter significance and misclassification error. Median viral gene copies quantified with RT-qPCR and RT-ddPCR were compared using Wilcoxon rank sum exact tests, and correlations between quantities from each method were compared using Pearson's product-moment correlation.

### Data availability.

All data are available upon request.
