# Supplementary material for: Rapid Assessment of SARS-CoV-2 Variant-Associated Mutations in Wastewater Using Real-Time RT-PCR
Source: Microbiol Spectr. 2023 Jan 11;11(1):e03177-22. doi: 10.1128/spectrum.03177-22 (PMC9927140; doi:10.1128/spectrum.03177-22)
Supplement: Supplemental file 1 — Supplemental material. Download spectrum.03177-22-s0001.pdf, PDF file, 0.4 MB [file spectrum.03177-22-s0001.pdf]

Supplementary material

for

Rapid assessment of variants of concern mutations in wastewater using real-time PCR

Kata Farkas[a,b,*], Cameron Pellett[a], Rachel Williams[a], Natasha Alex-Sanders[a], Irene Bassano[c,d], Mathew. R. Brown[c,e], Hubert Denise[c], Jasmine M.S. Grimsley[c,f], Jessica L. Kevill[a], Mohammad S. Khalifa[c,g], , Igor Pântea[a], Rich Story[c], Matthew J. Wade[c,e], Nick Woodhall[a], Davey L. Jones[a,h]

Kata Farkas[a,b,*], Cameron Pellett[a], Rachel Williams[a], Natasha Alex-Sanders[a], Irene Bassano[c,d], Mathew. R. Brown[c,e], Hubert Denise[c], Jasmine M.S. Grimsley[c,f], Jessica L. Kevill[a], Mohammad S. Khalifa[c,g], , Igor Pântea[a], Rich Story[c,h], Matthew J. Wade[c,e], Nick Woodhall[a], Davey L. Jones[a,i]

[a]Centre for Environmental Biotechnology, School of Natural Sciences, Bangor University, Bangor, Gwynedd, LL57 2UW, UK

[b]School of Ocean Sciences, Bangor University, Menai Bridge, Anglesey, LL59 5AB, UK

[c]UK Health Security Agency, Environmental Monitoring for Health Protection, Nobel House, 17 Smith Square, London, SW1P 3JR, UK

[d]Department of Infectious Disease, Imperial College London, London SW7 2AZ, UK

[e]School of Engineering, Newcastle University, Newcastle-upon-Tyne NE1 7RU, UK

[f]The London Data Company, London EC2N 2AT, UK

[g]Division of Biosciences, College of Health, Medicine and Life Sciences, Brunel University, London, UB8 3PH, UK

[h]Servita Professional Services (UK) Ltd, The Smiths Building, Great Portland Street, London, W1W 5PL

[i]Food Futures Institute, Murdoch University, 90 South Street, Murdoch, WA 6105, Australia

Table S1: generalised linear model with binomial residuals predicting NGS VoC detection using RT-qPCR variant-specific assays. The model results include the variable coefficient indicating its effect, followed by the standard error in brackets, and a significance code (p-value: < 0.001 [***]; < 0.01 [**]; < 0.05 [*]; > 0.05 [*NS*]). Pos, positive detection, Neg, negative detection.

Variables	Estimates
Intercept	-1.849 (0.071) ***
qPCR detection	2.553 (0.196) ***
AIC	1521.558
Misclassification error	0.151

qPCR	NGS	target	n
Neg	Neg	Beta	439
Neg	Pos	Beta	13
Pos	Neg	Beta	6
Neg	Neg	Delta	167
Neg	Pos	Delta	180
Pos	Neg	Delta	19
Pos	Pos	Delta	89
Neg	Neg	Gamma	435
Neg	Pos	Gamma	4
Pos	Neg	Gamma	19
Neg	Neg	Kappa	420
Neg	Pos	Kappa	33
Pos	Neg	Kappa	1
Pos	Pos	Kappa	2

target	+	negative	?	Total
Beta	13	446		459
Delta	266	189	4	459
Gamma	4	455		459
Kappa	18	424	17	459
				0
Total	301	1514	21	1836

target	FALSE	TRUE	Total
Beta	821	6	827
Delta	714	109	823
Gamma	808	19	827
Kappa	822	3	825
			0
Total	3165	137	3302

Table S2: generalised linear model with binomial residuals predicting NGS VoC detection using RT-ddPCR variant-specific assays. The model results include the variable coefficient indicating its effect, followed by the standard error in brackets, and a significance code (p-value: < 0.001 [***]; < 0.01 [**]; < 0.05 [*]; > 0.05 [*NS*]). Pos, positive detection, Neg, negative detection.

Variables	Estimates
ddPCR detection	0.31 (0.397) *NS*
AIC	59.607
Misclassification error	0.524

qPCR	NGS	ddPCR	target	n
Neg	Pos	Neg	Beta	3
Neg	Pos	Pos	Beta	3
Pos	Neg	Neg	Beta	1
Pos	Neg	Pos	Beta	3
Neg	Pos	Neg	Delta	2
Neg	Pos	Pos	Delta	6
Pos	Neg	Neg	Delta	1
Pos	Neg	Pos	Delta	2
Pos	Pos	Pos	Delta	6
Pos	Neg	Neg	Gamma	3
Pos	Neg	Pos	Gamma	6
Neg	Pos	Neg	Kappa	6

target	FALSE	TRUE	Total
Beta	6	8	14
Delta	5	17	22
Gamma	4	18	22
Kappa	6		6
			0
Total	21	43	64

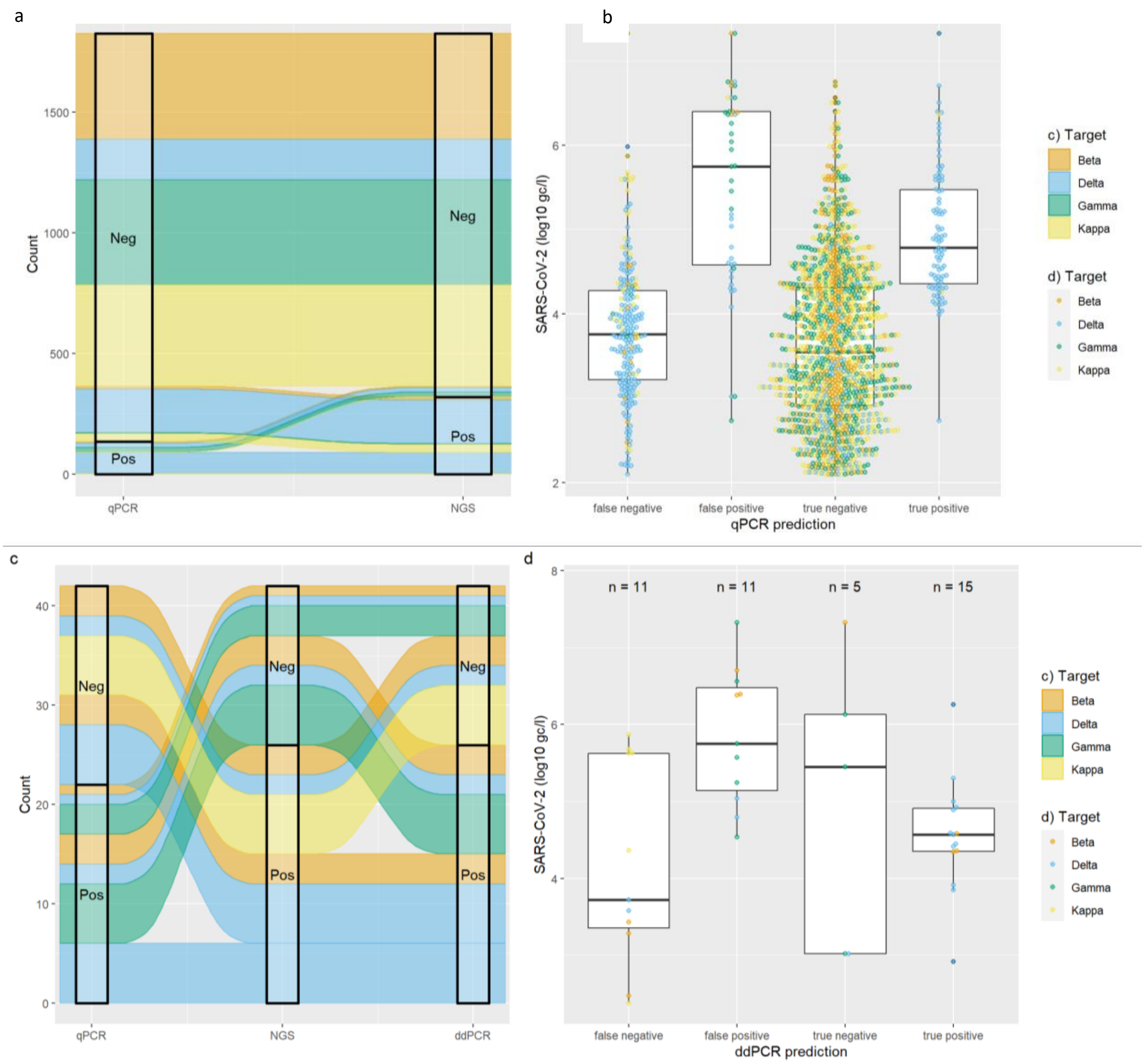

Figure S1. Panel ‘a’ shows an alluvial plot with the count of positive (Pos) and negative (Neg) detections of each of the VoC with qPCR and NGS. The counts are linked between qPCR and NGS to show the correct and incorrect correspondence in results between qPCR and NGS. Panel ‘b’ shows the results of a logistic regression predicting NGS of VoC using RT-qPCR detection on the x-axis and the $\log_{10}$ viral concentration (N gene) on the y-axis. Significant differences were found between groups (ANOVA: df = 3; F-value = 113.8; p-value < 0.001) and all pairwise comparisons (paired t-tests with p-values adjusted using the Holm-Bonferroni method: p-value < 0.05). Panel ‘c’ shows an alluvial plot with the count of positive (Pos) and negative (Neg) detections of each of the VoC with RT-qPCR, NGS and RT-ddPCR. The counts are linked between qPCR and NGS to show the correct and incorrect correspondence in results between all methods. Panel ‘d’ shows the results of a logistic regression predicting NGS of VoCs using RT-ddPCR detection on the x-axis and the $\log_{10}$ viral concentration (N gene) on the y-axis. Significant differences were found between groups (Kruskal-Wallis rank sum test: chi^2 = 10.99, df = 3, p-value < 0.05), but not between all pairwise comparisons, with false positives and true negatives (specificity), and true positives and false negatives (sensitivity) not having significantly different concentrations of N gene (pairwise Wilcoxon rank sum tests with p-values adjusted using the Holm-Bonferroni method: p-value > 0.05).

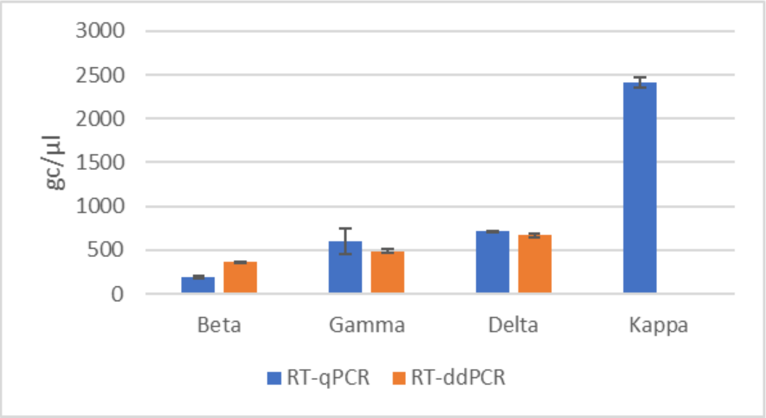

Figure S2. Concentration of PCR standards holding variant-specific mutations detected using RT-qPCR and RT-ddPCR. Values represent means± SEM.
